# The 23S Ribosomal RNA From *Pyrococcus furiosus* Is Circularly Permuted

**DOI:** 10.3389/fmicb.2020.582022

**Published:** 2020-12-10

**Authors:** Ulf Birkedal, Bertrand Beckert, Daniel N. Wilson, Henrik Nielsen

**Affiliations:** ^1^Department of Cellular and Molecular Medicine, University of Copenhagen, Copenhagen, Denmark; ^2^Institut für Biochemie und Molekularbiologie, Universität Hamburg, Hamburg, Germany; ^3^Genomics Group, Nord University, Bodø, Norway

**Keywords:** archaea, Pyrococcus, ribosomal RNA processing, circular permutation, fragmented ribosomal RNA

## Abstract

Synthesis and assembly of ribosomal components are fundamental cellular processes and generally well-conserved within the main groups of organisms. Yet, provocative variations to the general schemes exist. We have discovered an unusual processing pathway of pre-rRNA in extreme thermophilic archaea exemplified by *Pyrococcus furiosus*. The large subunit (LSU) rRNA is produced as a circularly permuted form through circularization followed by excision of Helix 98. As a consequence, the terminal domain VII that comprise the binding site for the signal recognition particle is appended to the 5´ end of the LSU rRNA that instead terminates in Domain VI carrying the Sarcin-Ricin Loop, the primary interaction site with the translational GTPases. To our knowledge, this is the first example of a true post-transcriptional circular permutation of a main functional molecule and the first example of rRNA fragmentation in archaea.

## Introduction

Synthesis and processing of rRNA is fundamental to all living organisms. A highly conserved feature at the transcriptional level is that the two main species, SSU and LSU rRNAs, are co-transcribed with the consequence that they are expressed directly into 1:1 stoichiometry. 5S rRNA on the other hand can be part of the same transcriptional unit or transcribed elsewhere in the genome. Another common principle applies to early pre-rRNA processing, namely that the 5´ and 3´ ends of each of SSU and LSU rRNA comes together as a prerequisite for their release from the ribosomal precursor. As a consequence, only fully transcribed precursors give rise to mature rRNA. In bacteria, the nucleotides corresponding to the mature rRNA ends as well as immediate flanking regions base pair to form stem structures that are recognized by RNase III, and the mature ends are subsequently formed by exonucleolytic trimming. In archaea, Bulge-Helix-Bulge (BHB) motifs are formed, cleaved by tRNA splicing endonuclease, and ligated by tRNA ligase to form circular intermediates. This is followed by further endonucleolytic cleavages and exonucleolytic trimming to form the mature rRNA ends. In the most studied models of eukarya (yeast and human), the primary transcript is not looped through base pairing and the initial cleavages are not coordinated in a single cleavage activity. Instead, multiple endonucleolytic cleavages and exonucleolytic trimming reactions result in the release of the mature rRNA species. The details of pre-rRNA processing and variations on the general schemes have been the subject of numerous reviews in bacteria (Deutscher, 2009), archaea (Clouet-d´Orval et al., 2018), and eukarya (Mullineux and Lafontaine, 2012; Henras et al., 2015). Considering the deep evolutionary conservation and central role in cell metabolism, it is fascinating that many variations in the topology of the mature rRNA can be found, including fragmentation, scrambling, and circularization.

Circularization of an RNA molecule followed by linearization by cleavage outside the circularization junction creates a circularly permuted RNA in which the transcriptional order of two continuous sequence elements AB is reorganized into BA. Additionally, such molecules are characterized by having a new sequence junction and a discontinuity compared to the co-transcriptional, linear counterpart. These structural differences may confer different biological properties to the circularly permuted molecular species. Circular permutation has been used as a method to study various properties of RNA molecules, e.g., the order of folding (Pan, 2000; Lease et al., 2007), including the folding (Kitahara and Suzuki, 2009) or tethering of rRNA (Fried et al., 2015; Orelle et al., 2015) and to make molecular tools, e.g., permuted group I introns as vehicles for producing circularized exons (Puttaraju and Been, 1992; Ford and Ares, 1994) or permuted rRNA in a protocol for the incorporation of non-natural nucleoside analogs (Erlacher et al., 2011). In nature, circular permuted RNAs can be produced by RNA processing or by genomic rearrangement. Any of the mechanisms that generate circular RNA (reviewed in Petkovic and Muller, 2015; Chen, 2016; Ebbesen et al., 2016; Panda et al., 2017) can in principle give rise to circularly permuted molecules, but their existence remains to be documented. RNA splicing is particularly potent in generating circular RNA, and the spliceosome can generate circular RNA by back-splicing or internal splicing of lariat intermediates that result from exon skipping reactions. The point of this circularization, however, appears to be the stabilization of the RNA and when the circle is re-opened, e.g., in the case of the miRNA sponge ciRS-7, the circularly permuted product is immediately degraded to liberate bound miRNAs (Hansen et al., 2013). Genomic rearrangements resulting in circularly permuted tRNA genes have been described from six species of algae and one archaeon (Soma, 2014). Here, the 3´ half is transcribed upstream of the 5´ half in a single transcript, that is, circularized and subsequently linearized to restore a conventional tRNA with functional ends. The processing is likely mediated by the tRNA intron splicing enzymes and it has been speculated that the permuted gene organization eliminates viral integration sites, similar to what has been proposed for fragmentation of tRNA genes by introns (Randau and Soll, 2008). In two species of the archaeon *Thermoproteus*, the SRP RNA gene is genomically rearranged, such that the transcription is initiated from a position that is normally found within the gene (Plagens et al., 2015). The precursor has short leader and trailers that form a BHB motif. This is processed by the tRNA splicing machinery to form a circular species that restores a fully functional SRP RNA. Here, the formation of a covalently closed molecule is seen as a mechanism to prevent unfolding of the RNA at extreme growth temperatures. These two latter examples have in common that the tRNA splicing machinery has been recruited to serve functions different from removal of introns.

Here, we demonstrate that the LSU rRNA of the extreme thermophile *Pyrococcus furiosus* is circularly permuted. Unlike the tRNA and SRP RNAs that are rearranged at the DNA level, the LSU rRNA in *P. furiosus* is transcribed as a conventionally organized LSU rRNA within a precursor encoding the SSU and LSU rRNA separated by a tRNA. The LSU is removed by a tRNA splicing-like mechanism that circularizes the LSU exactly at extended 5´ and 3´ ends. However, due to precise excision of helix 98 (H98), the mature LSU RNA appears as a linear RNA species that has helices 99–101 appended to the 5´ end and terminates in helices H94–97 comprising the Sarcin-Ricin Loop (SRL).

## Materials and Methods

### Cells Culture and RNA Extraction

*Pyrococcus furiosus* cells were grown in 1/2 SME medium (pH 6.5) supplemented with 0.1% yeast extract, 0.1% peptone, and 0.1% starch. The gas phase was N_2_/CO_2_ (80:20) and the incubation temperature was 95°C. The culture was grown for 30 h to a final cell density of 3 × 10^7^ cells/ml. RNA was extracted by resuspending 0.5 g of pelleted cells in 2.5 ml TMN buffer (50 mM Tris-Cl, pH 7.5, 10 mM MgCl_2_, and 100 mM NH_4_Cl). Cells were run twice through a French press, followed by addition of 4 ml TRIzol (Life Technologies). After 5 min incubation at room temperature, 0.8 ml chloroform was added. The samples were centrifuged at 12,000 × *g* for 5 min and the aqueous phase transferred to a new tube for extraction with phenol/chloroform. Total RNA was then precipitated by ethanol precipitation.

### Ribometh-Seq and Transcriptomics Analyses

The RiboMeth-seq analysis was performed in duplicates according to previously described protocols (Birkedal et al., 2015; Krogh et al., 2017). Briefly, the RNA was degraded by alkaline into short fragments and the 20–40 nt fraction purified from gels. Then, adaptors were ligated to the library fragments using a modified *Arabidopsis* tRNA ligase. Finally, the library was sequenced on the Ion Proton sequencing platform. The reads were mapped to non-coding RNAs annotated in the *P. furiosus* genome (GenBank: CP003685.1) and scored for read-end counts. Analyses of transcriptomics data were based on datasets deposited at the European Nucleotide Archive: SRX501747 (*Thermococcus kodakarensis*), SRX2118858 (*Pyrobaculum aerophilum*), SRX3467357 (*Sulfolobus acidocaldarius*), and SRX5547671 (*Pyrococcus furiosus*).

### Northern Blotting and Primer Extension

Northern Blot analysis was performed according to standard protocols as described in Josefsen and Nielsen (2010). The oligonucleotides used as probes were 5S rRNA: 5´-GGA TCG CTG GGG GGC TT, H98: 5´-GCC GGT CGC CCA GGC CCA, and H99–H101: 5´-GCA GGA CCT CGG GCG AT. AMV (Promega) or SuperScript IV (Invitrogen) reverse transcriptase was used in primer extension experiments according to the information provided by the supplier. Two oligos were used to map the 5´ end corresponding to the H98 excision site (oligo 1: 5´-GCA GGA CCT CGG GCG AT and oligo 2: 5´-ATC CCC GCC CTA TCA ACC GGG TCT T) and two for the conventionally assigned 5´ end (oligo 3: 5´-TAG CGT CCT AGC CCC TCT A and oligo 4: 5´-GGC GGC TTA GCG TCC TA). The RT-PCR experiment was made by first-strand cDNA synthesis of whole cell RNA using AMV reverse transcriptase (Promega) and dN_6_ primers, followed by standard PCR.

### Figure Making

RNA base pairing schemes were assisted by RNAfold from the ViennaRNA Web Services (http://rna.tbi.univie.ac.at/) and supported by covariance analysis of sequences from species of Thermococcales. Structure figures were prepared using PyMol and UCSF-Chimera.

## Results

### LSU rRNA in *Pyrococcus furiosus* Has Covalently Joined 5´ and 3´ Ends and Lacks H98

**Figure 1:**
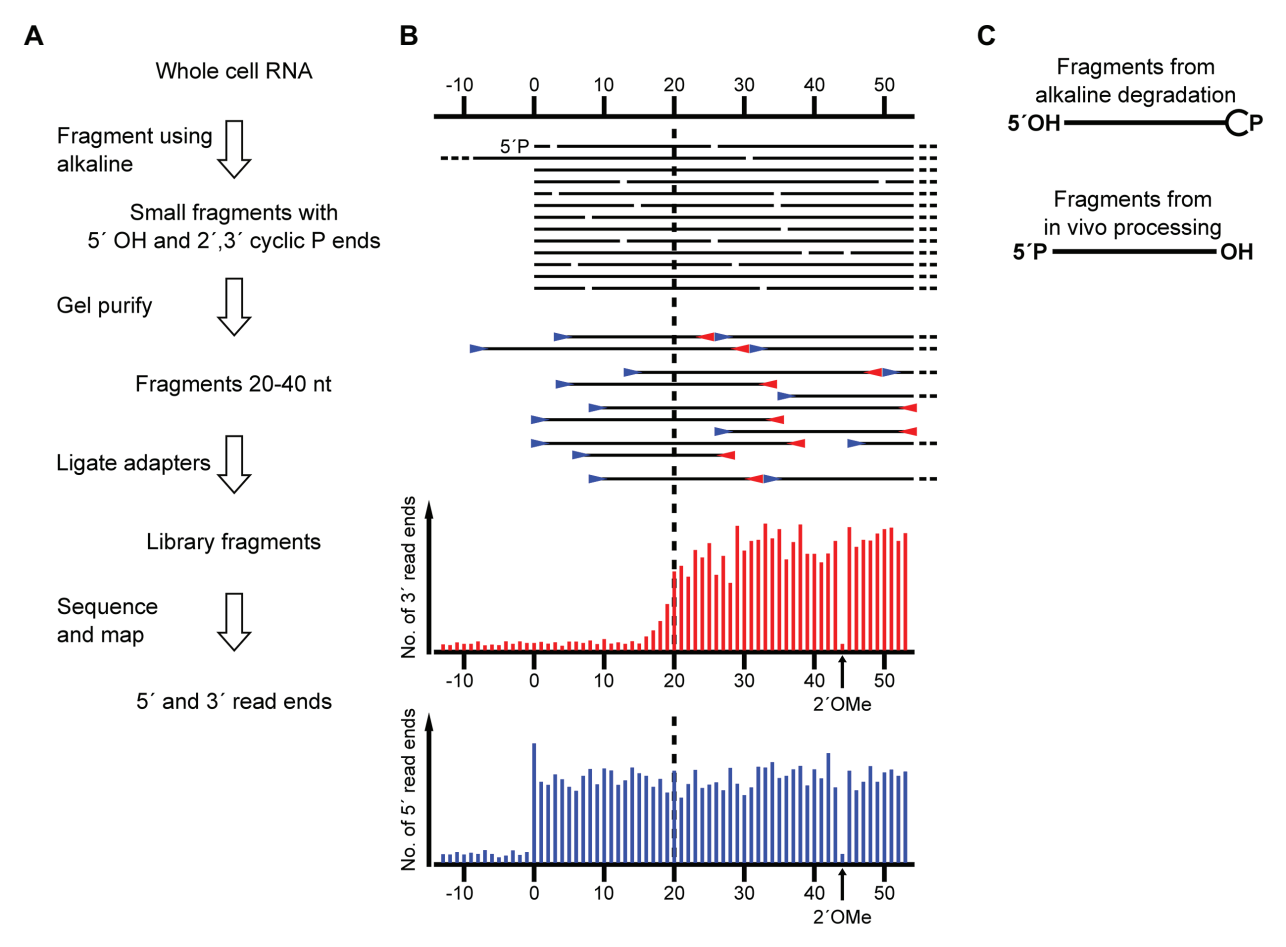
Schematic showing the application of RiboMeth-seq for analysis of native RNA ends. **(A)** Flow diagram of the steps in RiboMeth-seq. **(B)** Drawing of the fragments generated during the RiboMeth-seq protocol and the resulting read end coverage obtained at a native end. Note that the high number of read ends at the position corresponding to the native end as well as the depletion of 3´ read ends corresponding to the first 20 nt of the fragment carrying the native 5´ end. The depletion is caused by the loss of fragments <20 nt in length in the gel purification step. The signature resulting from a single 2´OMe in the RNA is indicated in the read end diagram. **(C)** Drawing highlights the end groups of internal fragments from alkaline degradation contrasted with RNA processing fragments from hydrolytic cleavages.

**Figure 2:**
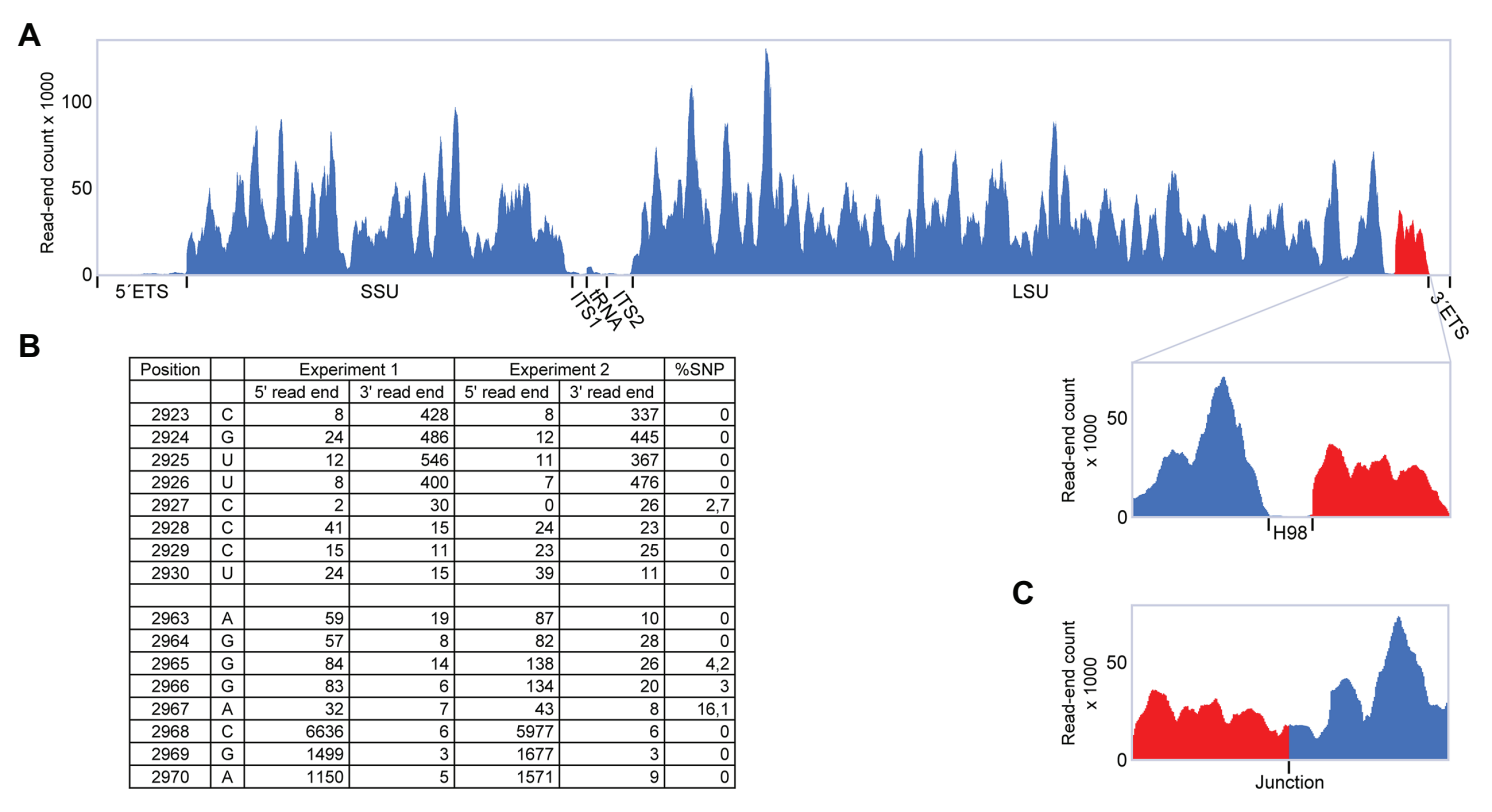
RiboMeth-seq analysis of *Pyrococcus furiosus* rRNA. **(A)** Read-coverage of the entire rRNA operon using a linear reference sequence. The 5´ and 3´ parts are in blue and red, respectively, to emphasize their rearrangement during circularization. **(B)** Table of actual read-end numbers at the borders of H98 showing the signature pattern for free ends at the H98 flanking regions. The fraction of single nucleotide polymorphisms (SNP) at read ends is shown in the rightmost column. **(C)** Read-coverage around the proposed circularization junction after re-mapping of the data in **(A)** using a circularized reference sequence.

During a RiboMeth-seq analysis of ribose methylation in *P. furiosus*, we observed several anomalies in sequencing read patterns. RiboMeth-seq is a method designed for profiling of ribose methylations in RNA (Birkedal et al., 2015; Krogh and Nielsen, 2019). In brief, it consists of partial alkaline fragmentation of RNA followed by cloning and sequencing based on the 5´ OH and 2´, 3´ cyclic phosphates generated by alkaline cleavage. 2´-*O*-Me protects against alkaline cleavage and, thus, methylated sites can be deduced from counting read-ends in the sequencing of library fragments. The RiboMeth-seq data on methylations in *P. furiosus* are deposited at the NCBI Gene Expression Omnibus database and will be reported elsewhere in the present article collection on archaeal ribosomes. Here, we focus on additional information provided by the RiboMeth-seq analysis on RNA organization. Each internal position of the RNA molecule being analyzed is covered by a 5´ read-end and a 3´ read-end corresponding to sequencing from either end of the library fragments. However, toward the ends of the molecule, reads are only obtained from one end due to a gel purification step in the protocol that removes fragments <20 nt (Figure 1A). Thus, 5´ and 3´ ends are recognized by depletion of 3´ or 5´ read-ends, respectively (Figure 1B). As a consequence, hidden breaks, e.g., as found in *Tetrahymena* LSU rRNA (Eckert et al., 1978), leave a distinct feature. Furthermore, ends that carry 5´ phosphates result in chimeric reads during cloning (Figure 1C), whereas 5´ OH ends do not. In this way, it is possible to distinguish ends originating from the two main types of cleavages, cleavage by transesterification and hydrolytic cleavage. Finally, circularization junctions can be extracted from reads as with any other RNA-seq method. During the analysis of *P. furiosus* rRNA, we observed very low coverage of nucleotides 2,927-2,967 corresponding to LSU H98 (Figure 2A) as well as the characteristic 5´ and 3´ read-end features signifying free ends (Figure 2B). Moreover, the many apparent single-nucleotide polymorphisms (SNPs) at nucleotides preceding C2968 (Figure 2B) suggested a large fraction of chimeric reads that were subsequently confirmed by inspection of actual reads. The chimera had 5´ parts originating from random fragments in the sample and thus derived from ligation during the cloning of library fragments demonstrating that C2968 carried a 5´ phosphate unlike the fragments generated by alkaline cleavage. Thus, H98 (41 nt) appeared to be cleanly excised at its base by a hydrolytic cleavage at the A2967-C2968 junction and likely by a similar reaction at the U2926-C2927 junction although we cannot exclude other mechanisms, e.g., removal of H98 by a combination of endonucleases and exonucleases. Chimeric reads from the latter cleavage reaction were probably depleted because cleavage fragments carrying the 5´ phosphate would be too small to be recovered in the protocol´s gel purification step. Excision of H98 should leave a 3´ LSU rRNA fragment of 129 nt. Due to failure to recover reads corresponding to the annotated ends of LSU rRNA during RiboMeth-seq analysis, we suspected that the terminal 129 nt was appended to the 5´ end. We then remapped all reads to a reorganized cyclic reference sequence. This resulted in consistent mapping with thousands of reads spanning the C3096-G1 junction (Figure 2C and Supplementary Figure S1A). Thus, we conclude from RiboMeth-seq analysis that the predominant form of LSU rRNA in *P. furiosus* is circularly permuted.

### LSU Circularization Involves a BHB Motif and Is Conserved Among Thermococcales

**Figure 3:**
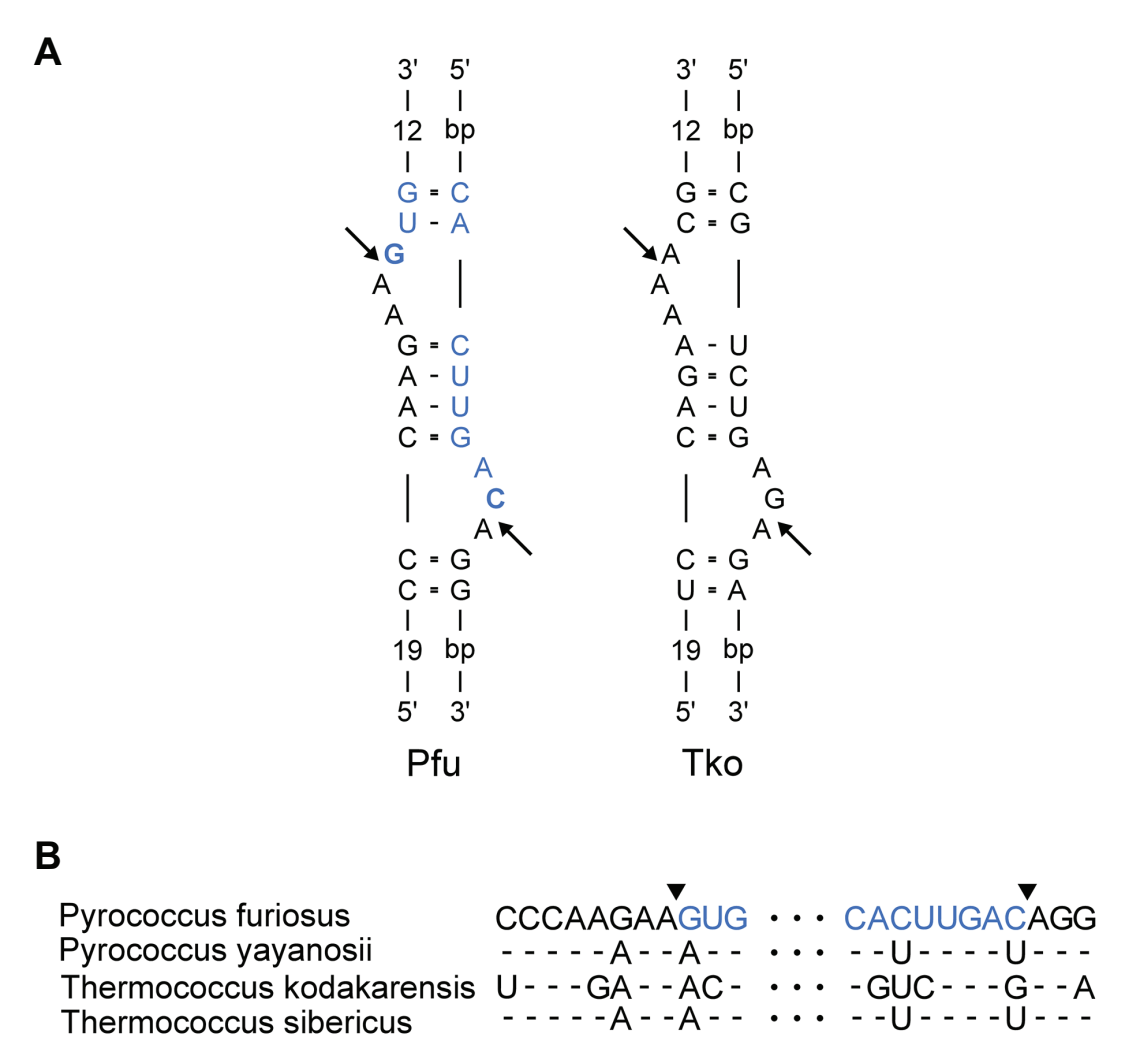
**(A)** Bulge-Helix-Bulge (BHB) motif involved in processing of *Pyrococcus furiosus* LSU rRNA. The arrows indicate canonical cleavage sites that will result in circularization of the rRNA. The mature rRNA in *Pfu* is colored in blue. The nucleotides that are joined to form the circularization junction in *Pfu* are in bold. Pfu: *Pyrococcus furiosus*. Tko: *Thermococcus kodakarensis*. **(B)** Alignment of sequences in the LSU BHB motif in species of Thermococcales. Arrowheads and color coding in the *Pfu* sequence corresponds to **(A)**.

Next, we inspected the *P. furiosus* pre-rRNA sequence for features that could underlie the proposed processing reactions. LSU rRNA and flanking sequences can form two stretches of extensive base pairing separated by a canonical BHB motif that presents the mature LSU rRNA 5´ and 3´ ends for cleavage and subsequent ligation by the tRNA splicing machinery (Figure 3A). The BHB motif is composed of a 4-base pair stem flanked by two 3-nt bulges (thus, a 3-4-3 BHB motif). The BHB motif and flanking helices are conserved among Thermococcales, e.g., in *T. kodakarensis* (Figures 3A,B). Cleavage of a BHB motif is catalyzed by tRNA splicing endonuclease and occurs at symmetrical positions within the bulges, resulting in 5´ OH and 2´, 3´ cyclic phosphate ends that are subsequently joined by a ligase, presumably a tRNA ligase. This type of processing of LSU rRNA has been proposed in other archaea. However, in *P. furiosus*, the mature ends become joined in contrast to the situation in, e.g., *A. fulgidus* and *S. solfataricus* (Tang et al., 2002), where nucleotides in flanking regions are joined and the mature ends are subsequently formed by endonucleolytic cleavage and exonucleolytic trimming reactions to form free 5´ and 3´ ends. The organization in *P. furiosus* further suggested that the flanking sequences became joined to generate a linear ITS2-3´ ETS molecule and, indeed, a junction demonstrating the existence of such a molecule could be detected among the reads (Supplementary Figure S1B).

In contrast to the LSU rRNA, the SSU rRNA appeared to be processed according to the conventional scheme. The 5´-ETS and ITS1 can co-fold to form a BHB motif separating two extended helices of 13 and 19 bp, respectively. The predicted cleavage sites are located 62 nt upstream of the mature SSU rRNA 5´ end and 27 nt downstream of the mature SSU rRNA 3´ end. The circularization sites creating a circular SSU rRNA intermediate and a chimeric 5´-ETS-ITS1 molecule could both be identified as low abundance reads, as expected. Specifically, we counted 95 and 42 reads, respectively, for the two junctions derived from SSU rRNA processing compared to 2,319 reads for the LSU rRNA circularization junction and 11 reads for the ITS2-3´ ETS junction. Further processing to form the mature SSU rRNA ends supposedly involves the action of endonucleases and exonucleases (Tang et al., 2002).

### Circularization and H98 Excision Are Supported by Northern Blotting and Primer Extension Experiments

**Figure 4:**
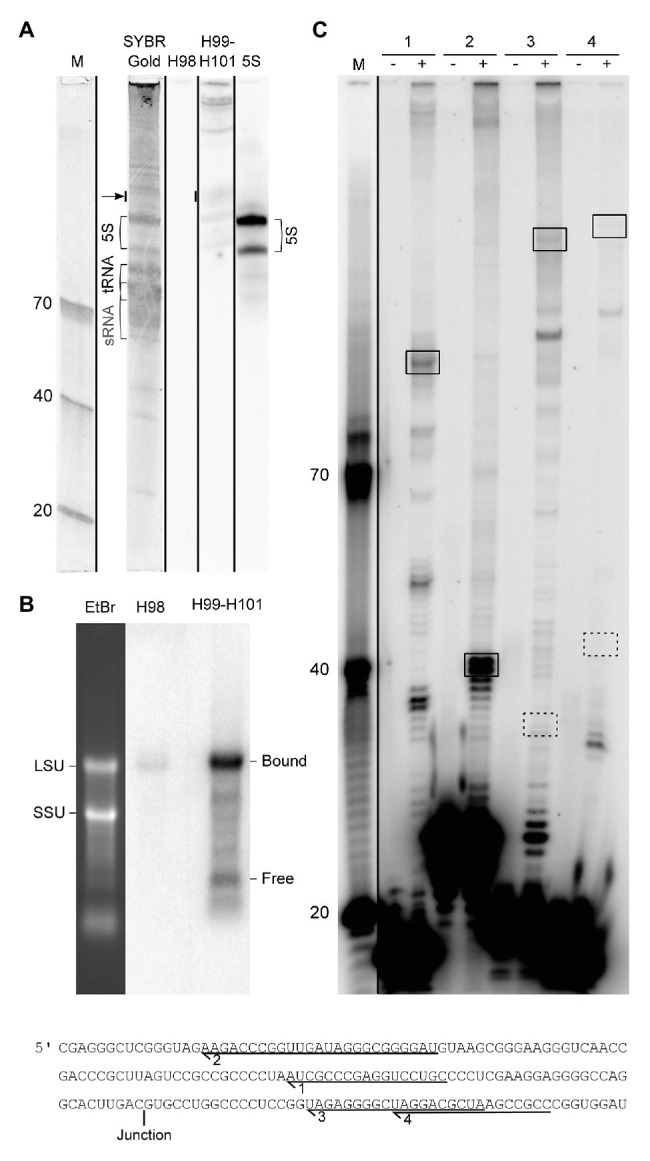
Experimental analysis of LSU rRNA circularization and H98 removal. **(A)** Northern blot analysis of the small RNA fraction of whole cell RNA fractionated on a 10% UPAG. The DNA oligonucleotide size marker (M) is followed by a SYBR Gold stained lane with indications of sRNA, tRNA, and 5S rRNA, followed by parallel hybridizations using probes against H98, H99–H101, and 5S rRNA (control). The lanes are separated by black lines to indicate that they were parallel lanes from the same gel. The arrow indicates the signal that was used to estimate the substoichiometric (compared to 5S rRNA) amounts of free 3´ terminal LSU rRNA fragment (129 nt; comprising H99-H101). The two forms of 5S rRNA detected may represent the two copies of the gene annotated with slightly different lengths in the genome browser (Chan et al., 2012). **(B)** Northern blot analysis of whole cell RNA fractionated on a 1% FA-agarose gel analyzed in parallel with H98 and H99–H101 probes. The positions of SSU and LSU rRNA, respectively, are marked on the gel stain, and the positions of bound (circularized) and free 3´ terminal LSU rRNA fragment (corresponding to H99-H101) are marked on the northern blots. **(C)** Primer extension analysis of the 5´ end of mature LSU rRNA. Comparison of the expected signals deriving from circularization and H98 removal (boxed with full lines) and conventional processing as inferred from the literature (boxed with dashed lines). The sequence in the lower part shows the location of the primers used in the experiment in relation to the circularization junction. (−) and (+) refer to addition of reverse transcriptase. M: DNA oligonucleotide marker.

In order to experimentally validate the proposed processing scheme, we first performed northern blotting and primer extension experiments. We were unable to detect the excised H98 by northern blotting analysis of small RNAs (Figure 4A) and primer extension experiments (data not shown). Thus, we exclude the possibility that the H98 exists as a prominent free RNA compared to 5S rRNA that was used as a control. Instead, we detected substoichiometric amounts of H98 co-migrating with LSU rRNA in agarose gels (Figure 4B). We estimated H98 to be present at <1% of the amount of the LSU rRNA 3´ part that was used as a control. This is consistent with the low number of sequencing reads from H98 in RiboMeth-seq analysis. We conclude that H98 is absent from mature *P. furiosus* ribosomes but can be detected at low levels in processing intermediates or incorrectly processed precursors. In parallel, we analyzed for the presence of the 129 nt 3´ end of LSU rRNA (H99–H101) that would exist as a separate fragment after H98 excision in the absence of ligation of the 5´ and 3´ end of LSU rRNA. We detected the free 129 nt fragment at a level of <5% compared to 5S rRNA based on the hybridization analysis as well as a gel stain (Figure 4A). Northern blot analysis of an agarose gel revealed that the bulk of the 3´ end is associated with a conventional LSU rRNA species, as expected from circularization of the precursor transcript (Figure 4B). This demonstrated that the ligation step is slightly less efficient than the preceding endonucleolytic step, at least under the present growth conditions. Primer extension analysis confirmed the absence of a 5´ end of LSU rRNA corresponding to pos. 1 on the gene map (primers #3 and #4) and instead demonstrated a strong signal at pos. C2968 (all four primers) in accordance with the proposed idea of a circularly permuted LSU rRNA (Figure 4C).

### H98 Is Located at the Surface of the Large Subunit and Is Optional in Archaea

**Figure 5:**
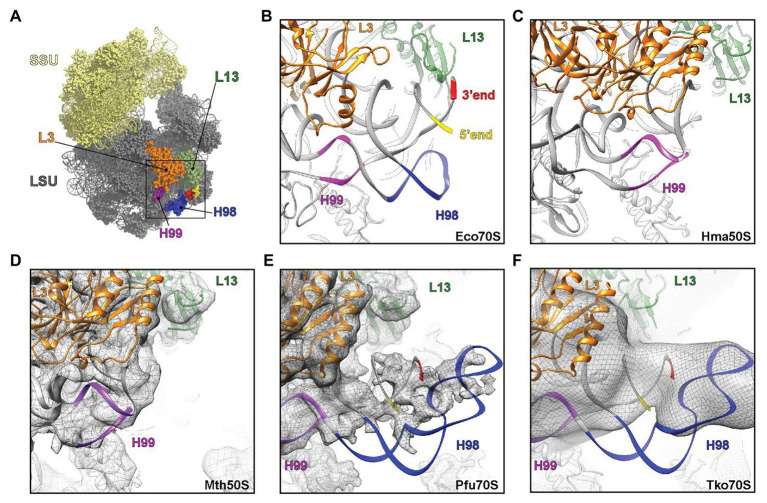
Visualization of H98 in structures of ribosomal particles from bacteria and archaea. **(A)** Overview of the *E. coli* ribosome with the SSU in yellow and the LSU in gray. Ribosomal proteins L3 (orange) and L13 (green) are highlighted for reference as are 23S rRNA helices H98 (blue), H99 (purple), and the 5´ (red) and 3´ (yellow) ends. **(B–F)** View of the large ribosomal subunit highlighting the region, where H98 (blue), H99 (purple), and the 5´ and 3´ ends are located in **(B,C)** the crystal structures of the **(B)** *E. coli* 70S ribosome (PDB ID 5GMP) and the **(C)** *Haloarcula marismortui* 50S subunit (PDB ID 4V9F), as well as cryo-electron microscopy structures of the **(D–F)** *Methanothermobacter thermautotrophicus* 50S subunit (EMD-2012, PDB ID 4ADX), **(E)** *Pyrococcus furiosus* 70S ribosome (EMD-2009, PDB ID 4V6U) and the **(F)** *Thermococcus kodakarensis* 70S ribosome (EMD-2170 with PDB ID 4V6U fitted). In **(D–F)**, the cryo-EM map density is shown as a gray mesh.

H98 is a highly variable helix that is nevertheless found in most organisms, including *Escherichia coli*, yeast, and humans. In bacteria, such as *E. coli*, H98 comprises 15 nucleotides that are located adjacent to the 5´ and 3´ ends of the 23S rRNA in the vicinity of ribosomal proteins L3 and L13 at the back of the large subunit (Figures 5A,B). By inspection of sequences deposited in the UCSC archaeal genome browser (Chan et al., 2012), the comparative RNA website (Cannone et al., 2002), and the full-length rRNA organismal alignment (FLORA) database (Doris et al., 2015), it appears that H98 is optional in archaea. It is generally found in species belonging to Crenarchaeota with occasional losses (e.g., in *Ignicoccus hospitalis* among the Desulforococcales), but absent in the two representatives of Nanoarchaeota, e.g., *Nanoarchaeum equitans*. In Euryarchaeota, it is found in, e.g., Methanococcales (with occasional losses, e.g., in *Methanococcus infernus*) and Thermococcales, but appears absent in, e.g., Methanomicrobiales, Halobacteriales, and Archaeoglobales (Supplementary Table S1). This is also confirmed by the X-ray crystal structure of the large subunit from the Halophile *Haloarcula marismortui* (Gabdulkhakov et al., 2013) and the cryo-EM structure of the 50S subunit from the methanophile *Methanothermobacter thermautotrophicus* (Greber et al., 2012), where H98 is replaced by a small linker of four nucleotides in both cases (Figures 5C,D). Our observations add further complexity to this picture, because we show that H98 is encoded within the Thermococcales, but subsequently excised and degraded.

Our observations of the sequence structure of *P. furiosus* LSU rRNA appear inconsistent with a published cryo-EM model of ribosomes at 6.6 Å resolution (Armache et al., 2013). Here, the cryo-EM data were modeled based on GenBank Acc. no. AE009950 and appears to lack 22 nt at the 5´ end and 25 nt at the 3´ end, respectively, compared to our sequence derived from rRNA sequencing with very high coverage. Moreover, re-examining the cryo-EM maps for the *P. furiosus* 70S ribosome reveals that the density in this region does not support the model proposed in the paper. Instead, the density would correspond with H98 being absent and the additional density being assigned to the extensions present at the 5´ and 3´ ends of the 23S rRNA (Figure 5E). Similarly, the examination of cryo-EM map of *T. kodakarensis* 70S ribosome at 16 Å resolution (Armache et al., 2013) also reveals the electron density that would be consistent with the absence of H98 and nucleotide extensions circularizing the 5´ and 3´ ends of the 23S rRNA (Figure 5F). Recently, high resolution (2.5-3.0 Å) cryo-EM structures of T. kodakarensis 70S ribosome were reported (Sas-Chen et al., 2020), where structural models for H98 are lacking due to the lack of density for H98. Re-analysis of the cryo-EM map, including low-pass filtering, revealed additional density extending from the 5´ and 3´ ends (Supplementary Figures S2A,B), analogous to that observed in the previous *T. kodakarensis* 70S ribosome structure (Armache et al., 2013). These observations are supported by re-analysis of existing transcriptomics data that confirm the circularization junction in mature LSU rRNA as well as the absence of H98 (Supplementary Figure S2). Thus, in conclusion, the cryo-EM density maps support the excision of H98 from the Thermococcales of the Euryarchaeota, consistent with the high-throughput sequencing data.

## Discussion

**Figure 6:**
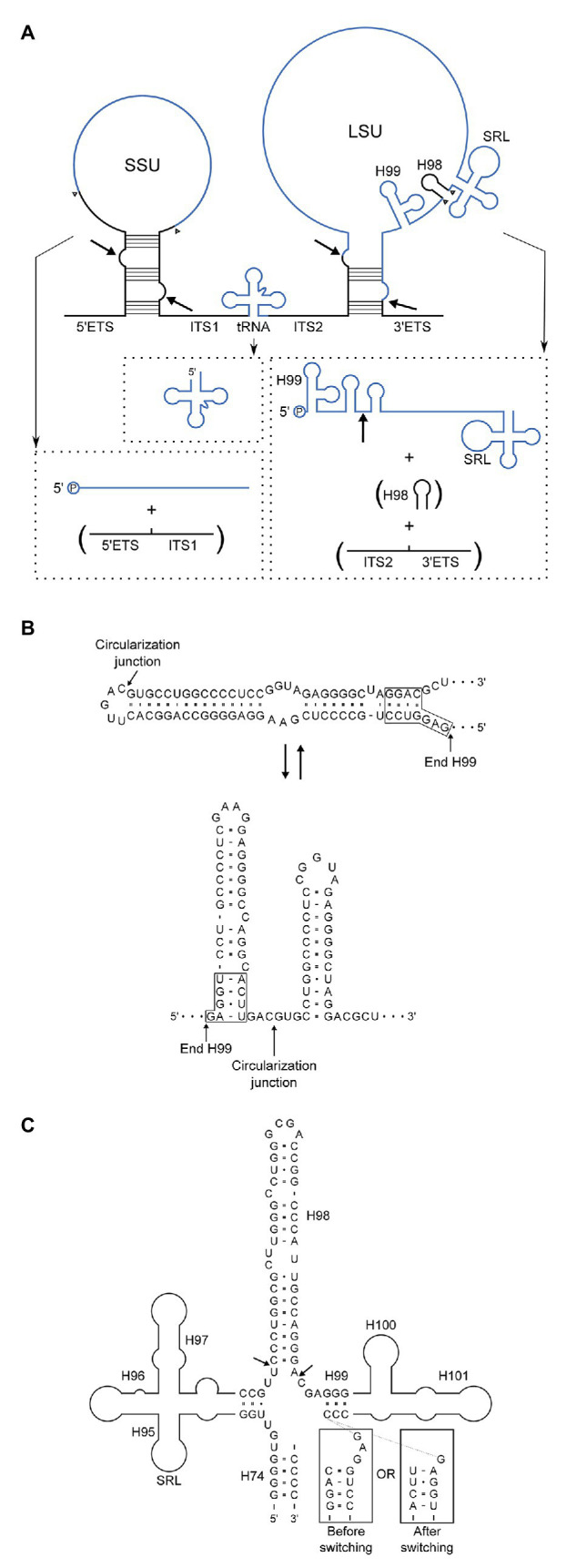
**(A)** Model of pre-rRNA processing in *Pyrococcus furiosus* including the rRNA precursor and final products. By-products that are turned-over are in parentheses. Intermediates, e.g., putative circular forms, were not included because the order of processing steps was not determined with certainty. Arrows indicate the proposed processing sites at BHB motifs and the circularization junction in the mature LSU rRNA, and arrowheads show the final processing sites for generation of the mature products. End-products are in blue and spacers in black. **(B)** Two alternatives base pairing schemes after circularization. The upper correspond to H1 in conventional annotation extended by 15 bp and closed by a loop. In the lower structure this helix is resolved into two helices spanning the circularization junction. **(C)** Structural context of H98 removal. H98 is located between H95 and H97 comprising the Sarcin-Ricin Loop (SRL) and H99–H101 involved in binding of the signal recognition particle (SRP). The arrows indicate the processing sites mapped by RiboMeth-seq analysis. The boxed sequences are similar to those in **(B)** and are appended to the 3´-strand of H99 to show how the alternative base pairing affects the structure of the junction at which H98 excision takes place.

We have shown by high-throughput sequencing as well as by northern blotting and primer extension that the main form of LSU rRNA in *P. furiosus* is circularly permuted. Based on our experiments, we propose a model for pre-rRNA processing in *P. furiosus* and other species of Thermococcales that is conventional with respect to formation of SSU rRNA as well as the tRNA located between the SSU and LSU rRNAs but has two unconventional features with respect to processing of LSU rRNA (Figure 6A). First, the LSU rRNA is released by a tRNA splicing-like mechanism exactly at its 5´ and 3´ ends. As a consequence of the release mechanism, the ends are covalently joined. Second, H98 is excised, likely by two coordinated hydrolytic cleavage reactions and subsequently degraded. Thus, LSU rRNA in *P. furiosus* exists as a circularly permuted RNA.

Circularization of RNA is widespread in archaea (Danan et al., 2012) and *P. furiosus* appears to be particularly prone to RNA circularization, e.g., with abundant circularization of box C/D RNAs (Starostina et al., 2004). Recently, an RNA ligase that ligates 5´-P and 3´-OH ends was characterized from *Pyrococcus abyssi* (the *Pab* 1,020 RNA ligase) and proposed to circularize many different cellular RNAs (Becker et al., 2017). The excision of H98 in *P. furiosus* curiously resembles a step in rRNA processing in chloroplasts. Here, the 3´ terminal part of LSU rRNA corresponding to helices 99–101 is encoded as a 4.5S rRNA that is separated from the bulk of the LSU rRNA by an internal transcribed spacer (Whitfeld et al., 1978), much like 5.8S rRNA that is encoded as the 5´ part of LSU rRNA in eukaryotes. The location of the internal transcribed spacer coincides with H98. In spinach chloroplasts, the spacer comprises 115 nt and can adopt a compact structure with a base-paired stem. Although the mechanism of removal of this spacer is unknown, it is formally very similar to H98 excision in *P. furiosus* ribosomes.

The circularization reaction and the excision of H98 are unlikely to be mechanistically coupled because they occur at distant sites and by different mechanisms. However, the reactions may be linked through a conformational switching mechanism (Nagel and Pleij, 2002). Prior to circularization, the BHB motif is flanked by two extraordinarily stable helices of 21 and 14 bp. The cleavage and ligation reactions remove the 21 bp lower of the two helices, and we speculate that the upper helix can now switch into a conformation in which the two strands fold back on themselves to form two independent helices flanking the circularization junction (Figure 6B). The two proposed alternative structures have roughly the same number of base pairs. Importantly, the conformational switch changes the structure of the junction from which H98 emerge (Figure 6C), and we speculate that only one of the two structures presents the base of H98 for cleavage. The structure of H98 and its flanking sequences as well as the possibility to undergo conformational switching upon circularization is conserved in *T. kodakarensis*. If circularization in this scheme precedes excision of H98, it should be possible to capture full-length circular molecules by RT-PCR. Unfortunately, this proved technically challenging. The opposite order of reactions, i.e., H98 excision prior to circularization, would lead to transient existence of two large cleavage fragments of the ribosomal RNA precursor. We did not detect such molecular species at sensitivity of analysis that would reveal rare to medium abundant mRNAs. Thus, the experimental discrimination between the two possibilities remains unsettled.

From an evolutionary point of view, the reorganization of LSU rRNA may have resulted from loss of a processing endonuclease that processed the circular intermediate following processing at the BHB motif. H1 is known to interact with H98 in archaea and the extended H1 or other structures resulting from the extended sequence (Figure 6B) may have structurally clashed with H98 driving evolution toward H98 elimination. In a recent paper on expansion segments in Asgard archaea (Penev et al., 2020), the *P. furiosus* is highlighted because it is supposed to have the longest H98 among archaea. H98 is the basis of ES39 that is “supersized” in Asgard archaea and of considerable length in eukaryotes. *Pyrococcus furiosus* carries a “μ-ES39” and its predicted structure presents a problem to the accretion model of rRNA expansion because it does not overlap in 3D structure when compared to *E. coli* in which H98 interacts within the H1 minor groove through an A-minor interaction. We propose instead that the lack of H98 provides space for an extended structure replacing H1, which is opposite to eukaryotes in which H1 is lacking giving way for a much expanded H98-ES39.

A recent, comprehensive review described the endoribonucleases and exoribonucleases known from archaea (Clouet-d´Orval et al., 2018). Whereas the enzymes responsible for the circularization step most likely are the tRNA splicing enzymes, the enzyme(s) responsible for H98 excision are unknown. The present data provide two clues to the activity. First, the observation of chimeric reads in RiboMeth-seq suggests that cleavages occur by hydrolysis leaving 5´-phosphate and 3´-OH. Second, the conformational switching model suggests that a particular structure at the cleavage junction may be induced. Furthermore, the excision removes a double stranded region and leaves unpaired ends, which argue for an initial endonucleolytic cleavage followed by exonucleolytic degradation of H98 and structural protection of the mature ends. Several endonucleases involved in rRNA maturation are known from bacteria (Taverniti et al., 2011; Clouet-d´Orval et al., 2015; Vercruysse et al., 2016). Of particular interest, PPR-SMR proteins are responsible for some endonucleolytic cleavages in chloroplast rRNA processing (Zhou et al., 2017) and related proteins exist in archaea, but the activity specifically responsible for processing of the spacer upstream of 4.5S RNA, that resembles H98 removal, remains to be characterized. RNase III is sporadic in archaea (Nicholson, 2014) but a protein of unknown function comprising an RNase III domain has been annotated in *Pyrococcus*. Recently, the RNase E-like FAU-1 endonuclease from *Pyrococcus* and *Thermococcus* was shown to be involved in pre-5S rRNA processing (Ikeda et al., 2017). It was also observed that LSU rRNA in ΔFAU-1 cells is approximately 50 nt longer than wt LSU rRNA, roughly corresponding to the length of H98. However, there is no obvious resemblance between pre-5S rRNA and the invoked structures of the sequences flanking H98. Future experiments should be directed toward experimental characterization of the activity based on cell extracts and model RNA substrates as well as recent developments in *in vivo* characterization of pre-rRNA processing (Juttner et al., 2020).

Processing of LSU rRNA in archaea is not understood in full detail despite recent progress (Grünberger et al., 2019; Juttner et al., 2020; Qi et al., 2020). However, clues to the diversity in processing can be obtained from both RiboMeth-seq analyses and the existing transcriptomics data obtained for other purposes. From RiboMeth-seq studies, we conclude that species of Methanococcales as well as *I. hospitalis* and *N. equitans* have free 5´ and 3´ ends and are uninterrupted in the H97-(H98)-H99 region (data not shown). In addition to the circularly permuted forms in *P. furiosus* and *T. kodakarensis* (Figure 2 and Supplementary Figure S2), we find, by analysis of transcriptomics data deposited in the European Nucleotide Archive, that LSU rRNA from *P. aerophilum* is circularized at a BHB motif located at the mature ends and retains H98 (Supplementary Figure S3). *S. acidocaldarius* is well-studied and has a canonical BHB motif at a distance from the proposed mature LSU rRNA ends and are thought to employ endonucleases and exonucleases to further process the circular intermediate formed at the BHB motif (Tang et al., 2002). However, processing at these sites have not been characterized, and although coverage is relatively low in this region, we find more that 10,000 reads spanning the circularization junction and too few read ends to support free LSU rRNA ends in this region (Supplementary Figure S4), suggesting that this species is the mature form rather than a processing intermediate. The circularized species has previously been demonstrated as an abundant species (Danan et al., 2012; Juttner et al., 2020). H98 is clearly present, suggesting that LSU rRNA in *S. acidocaldarius* remains circular. However, this must await direct characterization by independent methods. It is a characteristic of LSU rRNA processing that the two ends come together at an early stage of ribosome formation. The covalent joining of the ends through rRNA circularization demonstrated in this study presents the most radical of strategies toward this.

Fragmentation of rRNA has not been reported from archaea but is not uncommon and can result from genome organization or rRNA processing. In the mitochondria of *Tetrahymena pyriformis*, the 5´ end of LSU rRNA is transcribed downstream of the main gene body giving rise to a discontinuous rRNA synthesized in an unconventional order (Heinonen et al., 1987). Many bacteria transcribe pre-rRNA that becomes fragmented through the removal of intervening sequences by RNase III cleavage and trimming by other RNases (Evguenieva-Hackenberg, 2005). RNA fragmentation has been proposed to regulate the ribosome concentration by allowing faster turn-over (Hsu et al., 1994). This type of fragmentation is reminiscent of the “hidden gaps” found in protozoans and insects (Gray and Schnare, 1996). More dramatic fragmentation through removal of several intervening sequences is seen in, e.g., *Euglena gracilis* (Schnare and Gray, 2011) and in the LSU rRNA of several Trypanosomes (Hernandez and Cevallos, 2014; Shalev-Benami et al., 2016; Zhang et al., 2016; Liu et al., 2017).

As a consequence of the processing reactions in P. furiosus, LSU rRNA is circularly permuted and has a dramatic re-organization of two key elements in the ribosome. First, helices 99–101 are appended to the 5´ end of the molecule. H100 (or its structural equivalent) is involved in docking of the signal recognition particle (SRP) in bacteria and chloroplasts (Bieri et al., 2017). Second, helices H94–97 in Domain VI comprising H95 with the Sarcin-Ricin Loop (SRL) are placed at the very 3´ end. The SRL is the primary site of interaction between the ribosome and translational GTPases during protein synthesis. One intriguing possibility is that these elements of the ribosome are incorrectly positioned prior to the excision of H98, such that this step could serve as a late quality control for release of functional ribosomes. Quality control steps in ribosome biogenesis based on RNA cleavage or RNA modification are widespread (reviewed in Mullineux and Lafontaine, 2012; Sloan et al., 2016). The reorganization of the LSU rRNA may also serve as an adaptation for growth at elevated temperatures. The circularization may ensure the association of the 5´ and 3´ ends during ribosome biogenesis or protect the ends in the mature ribosome from accessibility due to thermal melting, as suggested for archaeal SRP RNA (16; Plagens et al., 2015). Alternative mechanisms may serve to protect the ends originating from H98 excision. Cryo-EM structures of spinach chloroplast ribosomes reveal an interaction of the 5´ end of 4.5S RNA and the 3´ end of the upstream LSU rRNA stabilized by an N-terminal extension of uL13 (Bieri et al., 2017; Graf et al., 2017). Such an extension is not present on *Pyrococcus* L13, where other strategies may apply.

In conclusion, we have demonstrated the existence of a naturally occurring, circularly permuted, and functional rRNA in the thermophilic archaeon, *P. furiosus*. This organism is widely studied and the organization of its rRNA was not anticipated in previous studies. However, re-analysis of existing data, e.g. from (Grünberger et al., 2019; Supplementary Figure S5) support our model. It will be of interest to study the structural and functional consequences on the ribosome of this re-organization of rRNA, and its possible role in adaptation of this organism to existence in a harsh environment.

## Data Availability Statement

The datasets presented in this study can be found in online repositories. The names of the repository/repositories and accession number(s) can be found at https://www.ncbi.nlm.nih.gov/ and https://www.ncbi.nlm.nih.gov/geo/query/acc.cgi?acc=GSE153501. Coordinates for the mentioned cryo-EM structures can be found in the Protein Data bank under accession numbers 5GMP (*E. coli* 70S ribosome), 4V9F (*H. marismortui* 50S subunit), 4ADX, (*M. thermautotrophicus* 50S subunit), 4V6 (*P. furiosus* 70S ribosome), and 4V6U (*T. kodakarensis* 70S ribosome).

## Author Contributions

UB and HN planned the RiboMeth-seq and biochemical experiments that were conducted by UB. DW supervised the cryo-EM analysis. DW and BB prepared the structure Figures. HN wrote the first manuscript draft. All authors contributed to the article and approved the submitted version.

### Conflict of Interest

The authors declare that the research was conducted in the absence of any commercial or financial relationships that could be construed as a potential conflict of interest.
