# Supplementary material for: The 23S Ribosomal RNA From Pyrococcus furiosus Is Circularly Permuted
Source: Front Microbiol. 2020 Dec 10;11:582022. doi: 10.3389/fmicb.2020.582022 (PMC7758197; doi:10.3389/fmicb.2020.582022)
Supplement: Supplementary file 1 [file Data_Sheet_1.pdf]

Supplementary Material

A

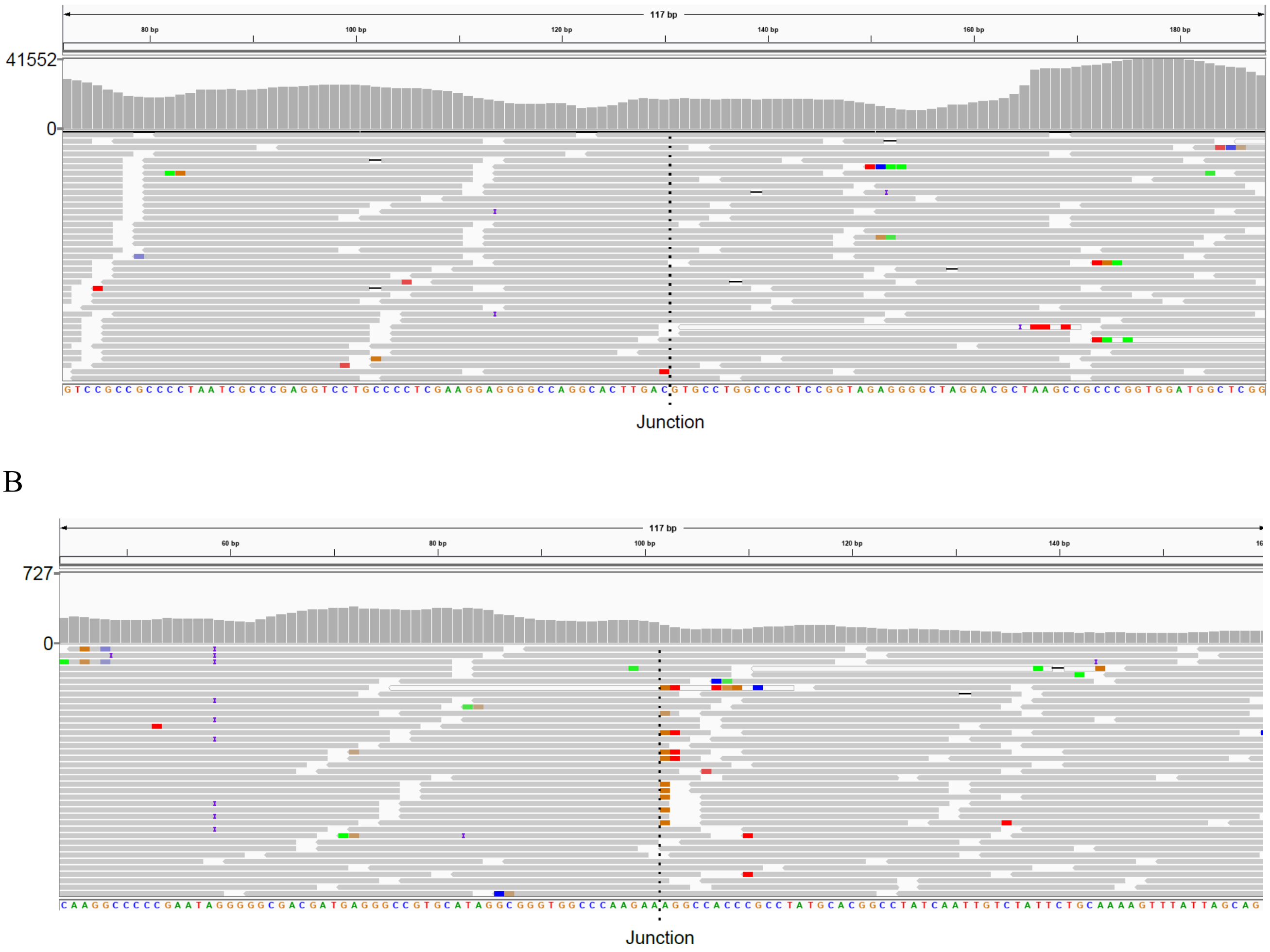

B

Figure S1. Diagrams showing sections of reads and total coverage (top panels) overlapping (A) the LSU rRNA circularization junction and (B) ITS2-3´-ETS in *P. furiosus.*

A-B

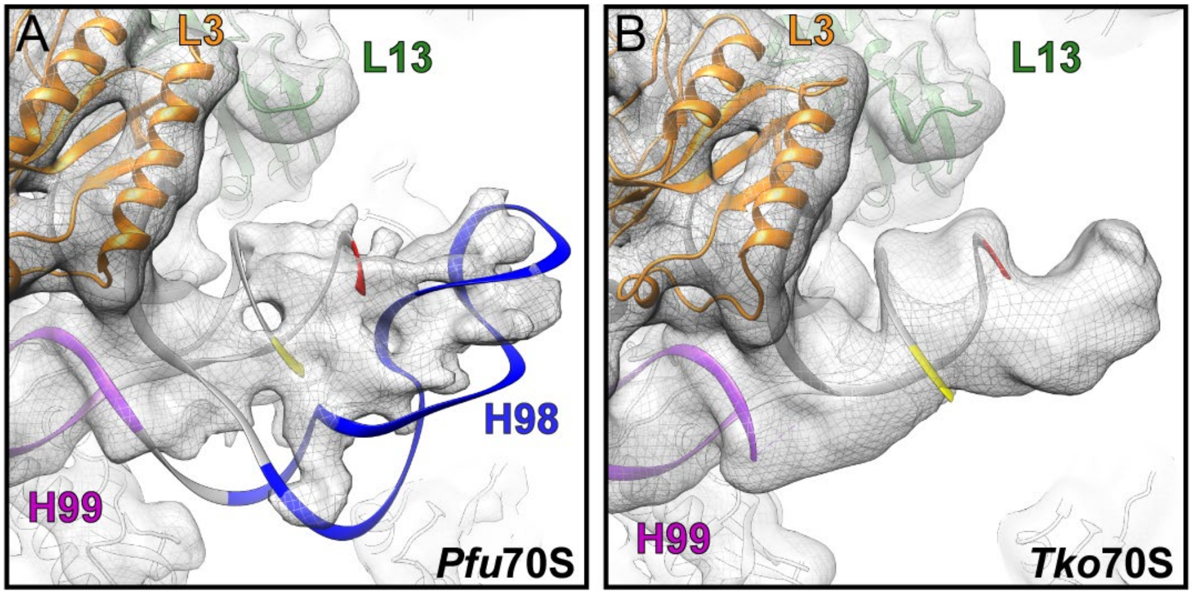

C

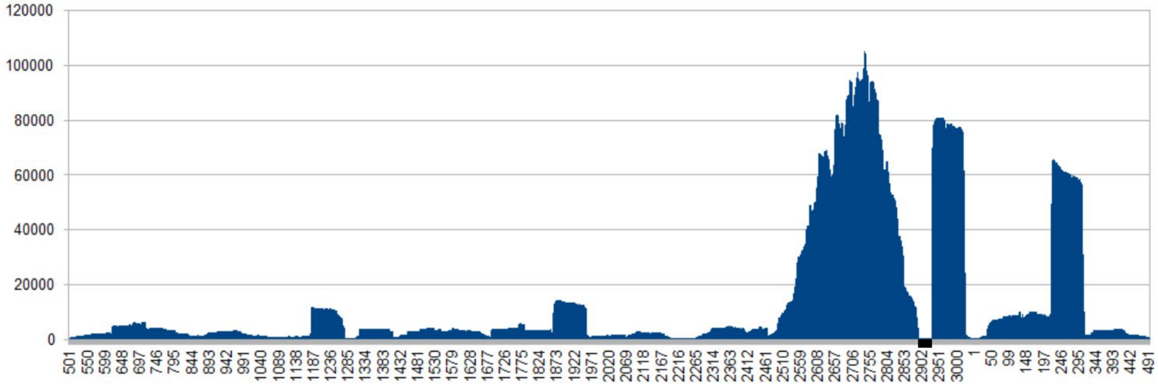

Figure S2. (A-B) View of the large ribosomal subunit highlighting the region where H98 (blue), H99 (purple) and the 5' and 3' ends are located in cryo-EM maps (grey mesh) of the *P. furiosus* (A; EMD-2009, PDB ID 4V6U) and *T. kodakarensis* (B; EMD-10223 with PDB ID 6SKF) 70S ribosome. Ribosomal proteins L3 and L13 are colored in orange and green, respectively. (C) Read-coverage in LSU rRNA from *T. kodakarensis* based on analysis of dataset ENA SRX501747 using a permuted reference sequence to examine the read-coverage across the putative circularization junction. The coverage landscape is highly biased due to treatment of the RNA with a 5´exonuclease (Terminator) and the use of a cloning kit for miRNA. However, H98 (marked with black square) has very low coverage and is positioned between the two main peaks in the diagram suggesting removal of H98. Furthermore, 282 reads were found to span the circularization junction suggesting a processing of LSU rRNA similar to that of *P. furiosus*.

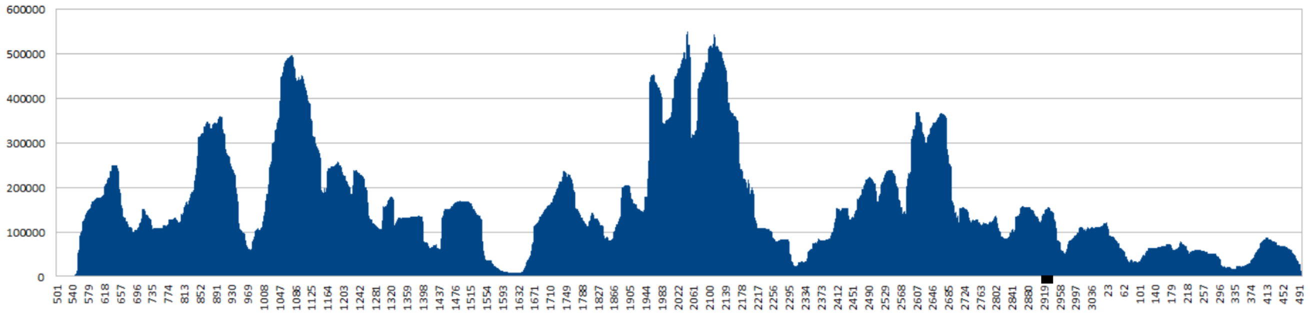

Figure S3. Read-coverage in LSU rRNA from *P. aerophilum* based on analysis of dataset ENA SRX2118858 using a permutated reference sequence to examine the read-coverage across the putative circularization junction. 100649 reads span the joined 5´and 3´ends suggesting that the LSU rRNA is circularized. H98 (marked with black square) is clearly present with coverage per nucleotide in excess of 130.000 reads.

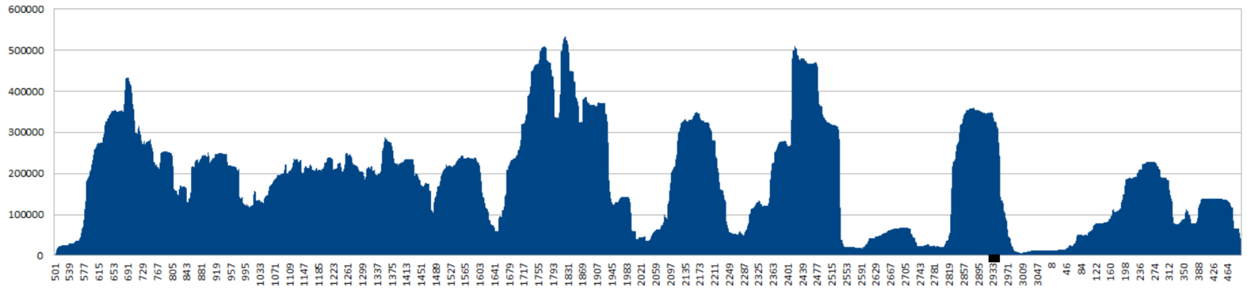

Figure S4. Read-coverage in LSU rRNA from *S. acidocaldarius* based on analysis of dataset ENA SRX3467357 using a permutated reference sequence to examine the read-coverage across the putative circularization junction. The annotated mature ends are represented by very few (<20) read ends. On the other hand, 11800 reads span the joined ends. H98 (marked with black square) is covered by >250.000 reads per nucleotide.

A

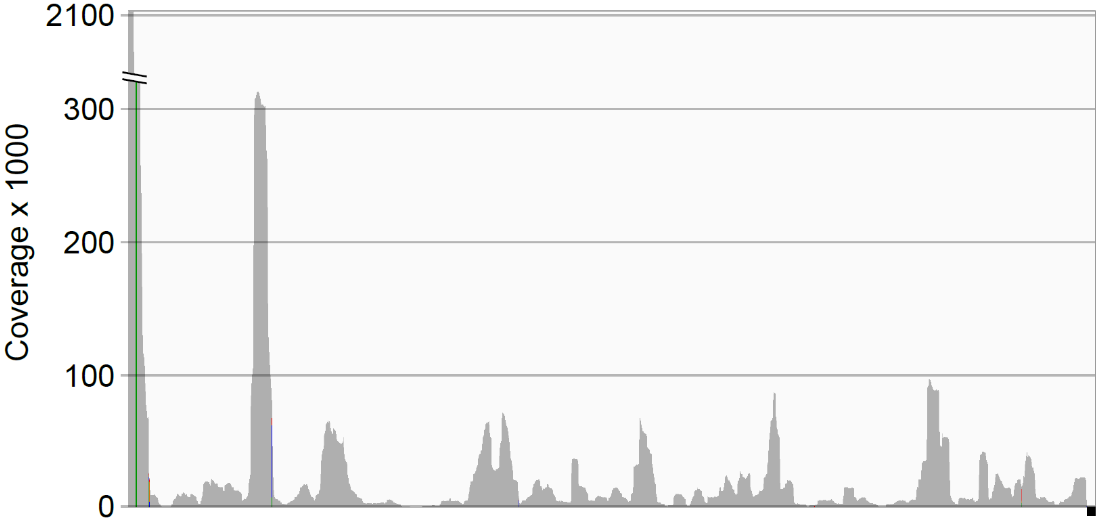

B

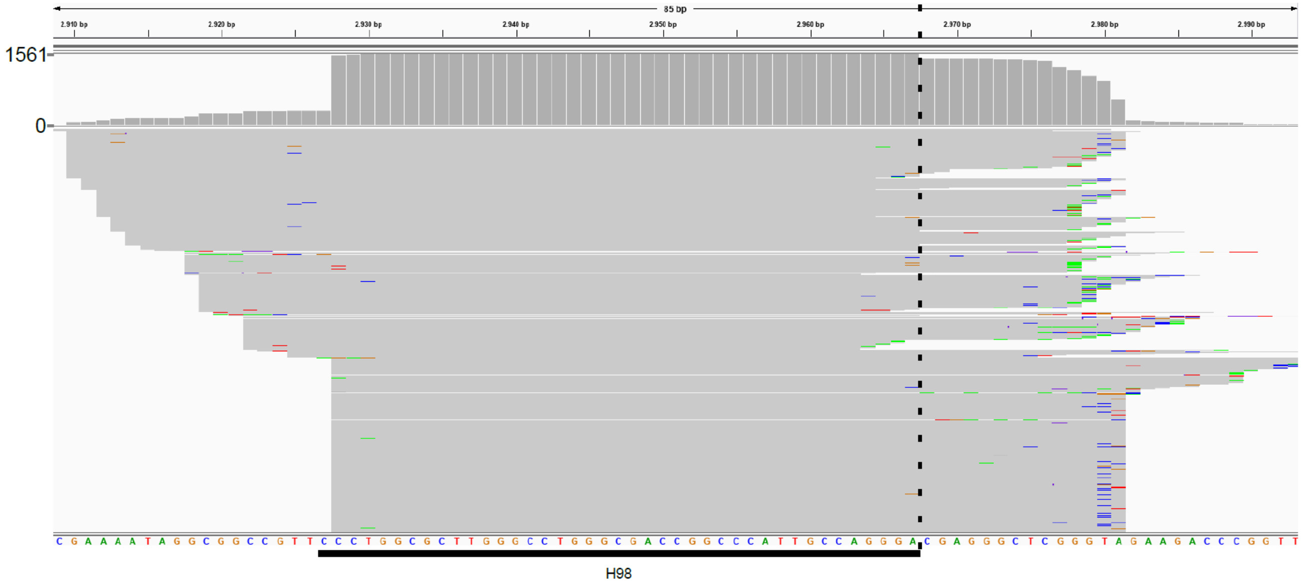

Figure S5. Read-coverage in LSU rRNA from *P. furiosus* based on analysis of dataset ENA SRX5547671 (Illumina sequencing of unfragmented RNA). Adapter sequence was trimmed from the reads using Cutadapt 2.8 with the sequence AGATCGGAA as input. Reads without adapter and reads shorter than 20 bp were discarded. (A) The remaining reads were mapped to the mature rRNA sequences using Bowtie2. The coverage is very uneven as expected from the protocol, and highlights the mature 5’-end corresponding to pos. C2968 that is grossly overrepresented. H98 (marked with black square) has very low coverage. (B) Reads containing the middle 33 nucleotides of H98 flawless (GGCGCTTGGGCCTGGGCGACCGGCCCATTGCCA) were selected (N=1731) and mapped to the precursor reference. The vast majority of (the relatively few) reads spanning this sequence, either 1) originates upstream of H98 and proceeds beyond H98 representing uncleaved precursor rRNA, or

2) originates at the 5’-end of H98 proceeds beyond the 3’-end representing precursor rRNA only cleaved at one end. Very few reads cover only H98 end-to-end in accordance with other methods failing to detect free H98.

Euryarchaeota
Methanomicrobiales
Methanospirillum hungatei
AUUAAA
Methanothermobacter thermoautotrophicus
UUGUGG

Halobacteriales
Haloarcula marismortui GCCGAG
Halobacterium halobium ACUGAG
Halobacterium volcanii CACUGAG

Thermoplasmatales
Thermoplasma acidophilum
GUCAUCAG

Archaeoglobales
Archaeoglobus fulgidus CAUUAAG

Methanococcales
Methanococcus vanniellii
CGU[UUCCUUCGGGAA]CGAG
Methanotorris igneus CAG[UCCCUUCGGGGA]CGAG
Methanocaldococcus jannaschii
CAG[UCCCUUCGGGGA]CGAG
Methanococcus infernus CAUGG

Thermococcales
Thermococcus celer CAC[UCCCAGGCGCAGGGGGUCGGGCGACCGGCCUUUGCCUGGGA]ACGAG
Thermococcus kodakarensis
ACU[CCUGGGCGUUGGGCCUGGGCGACCGGUCCGUUGCCCAGG]ACGAG
Pyrococcus furiosus GUU[CCCUGGCGCUUGGGCCUGGGCGACCGGCCCAUUGCCAGGG]ACGAG

Methanopyrales
Methanopyrus kandleri GUUGAG

Crenarchaeota
Desulforococcales
Desulforococcus mobilis
CUG[CCGUCACGGGUUACCGUGGCGG]CGCG
Desulfurococcus kamchatkensis
C[UGUCGUCACGGGGUAACCCGUGGCGGCG]CGGG
Ignicoccus hospitalis CAUUGAG
Staphylothermus marinus
CGU[CCGCCGAAGGGGUUUCCUCUUCGGCGG]GUAG
Hyperthermus butylicus C[GCCAGCCCGGGGCCACCCGGGCUGGC]CCGGG
Aeropyrum pernix CGUU[GGGCGCCGGCCUGGUGCCGGCGUCC]ACGAGGG

Sulfolobales
Sulfolobus acidocaldarius
CA[GGCGUUAGCC]GGGG

Thermoproteales

Thermoproteus tenax CGU[UGCCCUGGGGGGCAACCCCUGGGGCA]CGAG
Thermophilum pendens CAC[UCCCGAUCCGUUGCUCCCGGCGACGGGGGCUUUGGGUCGGGA]CGAG
Pyrobaculum aerophilum CGU[UGCCCUGGGGGCAACCCCGGGGCA]CGAG

Nanoarchaeota

Nanoarchaeum equitans GAUC

Table S1. List of H98 sequences from species representing major orders of Archaea. The last nucleotide in H97 and the first nucleotide of H99, respectively, are underlined. Nucleotides assigned to H98 are in [].
